# Recent advances in understanding and managing hepatic encephalopathy in chronic liver disease

**DOI:** 10.12688/f1000research.22183.1

**Published:** 2020-04-29

**Authors:** Annarein J. C. Kerbert, Rajiv Jalan

**Affiliations:** 1 Institute for Liver and Digestive Health, University College London, Royal Free Campus, Rowland Hill Street, London, NW3 2PF, UK

**Keywords:** Hepatic encephalopathy, hyperammonemia, inflammation, chronic liver disease

## Abstract

Hepatic encephalopathy (HE) is a common, severe complication of advanced chronic liver disease (CLD) and has a devastating impact on the patient’s quality of life and prognosis. The neurotoxin ammonia and the presence of systemic and neurological inflammation are considered the key drivers of this neuropsychiatric syndrome. Treatment options available in routine clinical practice are limited, and the development of novel therapies is hampered owing to the complexity and heterogeneity of HE. This review article aims to outline the current understanding of the pathomechanisms of HE and the recent advances in the identification and development of novel therapeutic targets.

## Introduction

In patients with advanced liver disease, liver insufficiency and/or portosystemic shunting may lead to the occurrence of a wide range of neuropsychiatric symptoms ^1^. This brain dysfunction, known as hepatic encephalopathy (HE), marks the end-stage of chronic liver disease (CLD) and has disastrous consequences for the quality of life of patients and their caregivers ^2, 3^. HE is a common feature in CLD, as it will develop in about 30–40% of patients at some point during the course of the disease. HE can be classified as overt (West Haven grade II–IV, diagnosed based on clinical symptoms ranging from disorientation to coma) or covert (minimal HE or West Haven grade I, diagnosis requiring specialist neuropsychologic testing [ Table 1]) ^1^. Overt HE development is unpredictable and rapid and often requires admission to the intensive care unit ^4, 5^. Prognosis of these patients is poor; unless there is access to liver transplantation, 1-year survival generally does not exceed 40% ^6, 7^. Also, minimal HE (mHE) is associated with a significant impact on quality of life and an increased risk of development of overt HE, hospital admission, and death. Despite advances in the understanding of HE and the development of novel therapies, recent data point out that it is still the leading cause for readmission and mortality in CLD ^5^.

**Table 1.** WHC and ISHEN classification (modified according to Vilstrup *et al*. ^1^).

WHC grade	ISHEN	Clinical features
Unimpaired		No present or previous HE
Minimal	Covert	Alterations of psychometric or neuropsychological tests (i.e. PHES, CFF, EEG) without clinical manifestations
Grade I		• Trivial lack of awareness • Euphoria or anxiety • Shortened attention span • Impairment of addition or subtraction • Altered sleep rhythm
Grade II	Overt	• Lethargy or apathy • Disorientation for time • Obvious personality change • Inappropriate behavior • Dyspraxia • Asterixis
Grade III		• Somnolence to semi-stupor • Responsive to stimuli • Confused • Gross disorientation • Bizarre behavior
Grade IV		Coma

CFF, Critical Flicker Frequency; EEG, electroencephalography; HE, hepatic encephalopathy; ISHEN, International Society for Hepatic Encephalopathy and Nitrogen Metabolism; PHES, Psychometric Hepatic Encephalopathy Score; WHC, West Haven Criteria.

Although it is well established that the neurotoxin ammonia is key in the pathogenesis of HE, the neurochemical changes following ammonia metabolism are numerous and not all yet fully understood. Moreover, distinct pathomechanisms such as alterations in cerebral blood flow (CBF) and, more recently, inflammation have been shown to contribute. The heterogeneity of the clinical presentation as well as the complex and multifactorial pathogenesis of HE have hampered the optimization of its management and the development of effective therapies. This article aims to review the most recent advances in understanding of the disease, with a focus on ammonia and inflammation, and the translation into clinical management. Moreover, the future perspectives of the most promising therapeutic opportunities will be discussed.

## Recent advances in understanding the complex pathophysiology

### Ammonia: the traditional hypothesis

Of all neurotoxin candidates that have been studied over the last century to explain the neuropsychiatric phenotype in liver disease patients, ammonia has been investigated and discussed most extensively. Ammonia is a nitrogenous compound that is mainly derived from bacterial production and amino acid metabolism in the gut ^8– 10^. In healthy individuals, ammonia is metabolized in the liver by the urea cycle and glutamine synthetase (GS) and subsequently excreted by the kidneys. The very first link between ammonia and HE dates back almost a century (1922), when a causal relationship between ammonia and meat intoxication was reported in dogs with a portocaval shunt ^11^. Later (1954), the significance of portal-systemic shunting in cirrhosis in the pathogenesis of HE was reported by Sheila Sherlock *et al*. ^12^. They measured peripheral and hepatic venous blood levels of ammonia in CLD patients following oral ammonium chloride intake and showed that ammonia can enter the systemic circulation via the gut by passing through a cirrhotic liver and/or by bypassing it via portal-systemic collaterals. This was therefore defined by the term “portal-systemic HE”. This circulating ammonia is then able to cross the blood–brain barrier (BBB), where it induces a cascade of deleterious effects on the brain ^13^.

### Ammonia: advances in understanding its cerebral effects

Once ammonia reaches the brain, its metabolism mainly relies on glutamine synthesis via GS, which is almost exclusively located in the astrocytes ^14, 15^. In physiological states, GS is already acting near its maximum rate, thereby efficiently converting ammonia into glutamine. However, in the setting of hyperammonemia, the brain becomes less efficient in ammonia removal because of an insufficient upregulation of GS activity and the absence of an alternative removal pathway ^16^. Nevertheless, glutamine concentrations are well known to be markedly increased in the brains of animals and patients with HE ^16– 18^. Inhibition of glutamine breakdown by ammonia has been suggested to be a contributing factor ^19^. Increased cytosolic glutamine creates an osmotic gradient and thereby contributes to the characteristic morphological changes and mild swelling of the astrocytes in chronic hyperammonemia, known as Alzheimer type II astrocytosis ^20^. Also, ammonia-related changes in the expression of key astrocytic proteins, such as glial fibrillary acidic protein ^21, 22^ and peripheral type benzodiazepine receptors ^23– 25^, contribute to the altered astrocyte morphology and dysfunction.

Besides low-grade brain edema and astrocyte dysfunction, HE in CLD seems to be characterized by a global (ammonia-induced) depression of the central nervous system’s function. This is reflected by 1) a net increase in inhibitory neurotransmission (mainly via impairment of the glutamate neurotransmitter system) ^26– 28^, 2) reduced CBF ^29, 30^, 3) reduced oxygen consumption and brain oxygenation ^31, 32^, and 4) reduced energy metabolism ^33, 34^. All of these factors seem to be closely interconnected, and imaging studies in cirrhotic patients with chronic hyperammonemia show similar redistribution patterns for CBF and the cerebral metabolic rate for glucose (CMR _glucose_), characterized by a decrease in the cortical and an increase in certain subcortical areas ^35^. This corresponds with regions of ammonia-induced suppression of brain metabolism and neurotransmission ^36^. The link between HE and impaired energy metabolism was first suggested in 1955 ^37^. Thereafter, studies showed that key processes such as glycolysis ^38– 40^, the tricarboxylic acid cycle (TCA) ^41, 42^, and the electron transport chain (ETC) ^43– 45^ are affected by ammonia. An increased rate of glycolysis is a well-characterized phenomenon in HE and hyperammonemia. Although increased glycolysis would be anticipated to increase the operational rate of the TCA cycle, this is not the case in hyperammonemia. Instead of being used in the TCA cycle, pyruvate produced during glycolysis is converted into lactate ^40, 46^. Diminished availability of pyruvate in hyperammonemia may lead to a decreased operational rate of the TCA cycle, ETC (reduced oxaloacetate generation and availability of NAD/NADH), and ultimately ATP production ^47^. Reduced brain ATP levels have been reported in experimental models of both acute and chronic hyperammonemia ^33, 48– 50^. Whether reduced brain ATP levels reflect suppressed synthesis or increased consumption is not yet fully elucidated. More recent studies have been focusing on the role of ammonia-induced mitochondrial dysfunction as an underlying mechanism of impaired energy metabolism in HE. Particularly, the mitochondrial permeability transition (mPT) has been suggested to play a central role. It is characterized by a sudden increase in permeability of the inner mitochondrial membrane to small molecules by opening of the permeability transition pore (PTP) ^51, 52^. The most important triggers of PTP opening are increased mitochondrial Ca ^2+^ and (ammonia-/glutamine-induced) production of reactive oxygen species (ROS) ^53^. This then leads to depolarization of the mitochondrial membrane potential, osmotic swelling of the mitochondrial matrix, uncoupling of the ETC, and thus inhibition of ATP synthesis. Ammonia has been shown to induce an early increase in intracellular Ca ^2+^ in cultured astrocytes and subsequent induction of mPT ^54– 57^. Although results of various *in vivo* and *in vitro* studies of acute and chronic hyperammonemia support the role of ammonia in disturbed energy metabolism and mitochondrial dysfunction, it must be noted that conflicting results exist ^47^. This may be partly explained by differences in studied brain regions, cell types, and ammonia concentration and durations. The chronological order of events and interrelationships among changes in neurotransmission, CBF, and oxygen and energy metabolism are yet to be clarified and may guide us to better understand this complex condition and ultimately to develop novel therapeutic strategies.

A recently opened field in the exploration of the pathogenesis of HE is ammonia-induced cellular senescence of astrocytes. It has been described that ammonia can induce senescence via glutamine synthesis-dependent formation of ROS, p53 activation, and upregulation of cell cycle inhibitors (p21 and GADD45a) ^58^. Another study describes a role for heme oxygenase (HO)-1 in mediating ammonia-induced inhibition of astrocyte proliferation in cultures ^59^. Although it is currently unknown whether there is a role for astrocyte senescence in the development of cognitive impairment in HE, it seems to have exciting implications for explaining the increasing evidence that cognitive dysfunction does not fully reverse in all patients who experienced an acute episode of HE and may even persist after liver transplantation.

### Ammonia: the refined hypothesis

The above-described selection of deleterious effects of ammonia on the brain form the basis of the traditional ammonia hypothesis. This is supported by the fact that ammonia-lowering therapies improve symptoms and outcome in HE, which are therefore the current cornerstones of therapy ^1^. However, this hypothesis is often criticized, mainly because the clinical value of ammonia measurements is, to date, still unclear, as plasma levels do not always correlate well with severity and outcome ^60^. This observation suggests that in different clinical situations the effect of ammonia on the brain may well be different. Features in cirrhosis such as inflammation, malnourishment, sodium levels, sarcopenia, co-morbidities, renal dysfunction/failure, and gastrointestinal bleeding (high intestinal protein load) may be some of the contributory factors.

### Systemic inflammation in chronic liver disease: role in hepatic encephalopathy

The poor correlation between circulating ammonia levels and HE severity in CLD led to the hypothesis that other mechanisms are involved. Systemic inflammation, commonly referred to as systemic inflammatory response syndrome (SIRS), is a common phenomenon in CLD and can occur in the context of non-sterile (i.e. bacterial infection) as well as sterile inflammation ^61^. It is characterized by the systemic release of pro-inflammatory cytokines (“cytokine storm”), which may subsequently culminate in severe impairment of systemic hemodynamics and organ hypoperfusion, organ inflammation, cell death, microvascular damage, and eventually (multi-) organ failure. It is well described that sepsis without underlying liver disease can present similarly to HE with altered mental state and motor function, a condition also referred to as “septic encephalopathy” ^62^. This indicates that a pro-inflammatory state itself can precipitate an encephalopathic state. Previous studies have shown that the vast majority of patients admitted with severe HE indeed present with evidence of systemic inflammation ^63^. Moreover, patients with CLD are generally immunosuppressed and therefore prone to infections, which are well-recognized precipitants of overt HE ^64^. The presence of systemic inflammation has been found to significantly impact on mortality risk, and pro-inflammatory markers correlate well with the severity of HE. Also, in patients with mHE, serum levels of pro-inflammatory cytokines are increased (IL-6, IL-18) and correlate with the degree of neurocognitive dysfunction and driving ability ^65, 66^.

Peripheral inflammation can lead to neuroinflammation via several pathways, of which the humoral (circulating cytokines) and immune (activated immune cells) pathways are the most important ^67^. Firstly, translocation of Gram-negative bacteria across the intestinal barrier and the release of bacterial products (i.e. pathogen-associated molecular patterns [PAMPs]) play an important role in the development of systemic inflammation in CLD ^68^. PAMPs, such as lipopolysaccharide (LPS), bind to pattern recognition receptors resulting in the release of pro-inflammatory cytokines. Circulating cytokines can directly enter the brain by impacting on the permeability of the BBB or by binding to receptors of pro-inflammatory cytokines (TNFα, IL1β) expressed by endothelial cells in the BBB ^69, 70^. Subsequently, this leads to the release of secondary messenger molecules into the brain. These molecules (such as prostaglandins and nitric oxide [NO]) can induce the activation of microglia that can themselves produce inflammatory mediators. Secondly, activated immune cells can similarly bind to endothelial cells in the BBB, thereby inducing the release of secondary messenger molecules and microglia activation. There is a high number of studies providing evidence of microglia activation in the brains of both rodent models and patients with HE. This neuroinflammatory state can lead to changes in neurotransmission, oxidative stress, and neuronal cell death, as shown in both *in vitro* and *in vivo* studies ^71– 73^.

Besides the alterations in intestinal integrity and increased bacterial translocation in CLD, an upcoming field in exploring the role of the gut–brain axis in HE is the gut microbiome. Considering the disrupted intestinal barrier and suppressed immune system in CLD, it is not surprising that dysbiosis of gut microflora can contribute to inducing peripheral inflammation in CLD. Characteristic changes in microbiome associated with HE have been found to correlate with cognitive function and systemic inflammation and involve an abundance of non-autochthonous microorganisms such as *Veillonellaceae, Alcaligenaceae, Enterococcus, Megasphaera, Burkholderia, Streptococcus salivarius, Staphylococcaceae, Porphyromonadaceae*, and *Lactobacillaceae* ^74– 76^. Similar findings have been reported in both stool and salivary microbiota. This suggests that a global gastrointestinal dysbiosis strongly correlates with cognition and inflammation in CLD and therefore holds prognostic and therapeutic potential in HE ^77, 78^.

### Systemic inflammation in chronic liver disease: synergy with ammonia

Increasing evidence points towards the fact that hyperammonemia and systemic inflammation are not two distinct mechanisms driving the severity of HE but that they are working synergistically by making the brain more susceptible to each other’s effects. An elegant clinical study by Shawcross *et al*. showed for the first time that scores of neuropsychological tests in stable cirrhotic patients are declining when hyperammonemia is induced in an inflammatory state but not after the infection has resolved ^79^. This synergism was later confirmed in animal models of CLD, showing that the administration of LPS results in hyperammonemia, brain swelling, and coma ^80, 81^. On the other hand, a reduction in blood ammonia was shown to protect the brain from a subsequent dosing of LPS, suggesting that not only does inflammation make the brain more susceptible to the effects of ammonia but also the reverse is true ^82^. Furthermore, hyperammonemia itself can directly induce microglia activation and neuroinflammation and appears to have a role in suppression of the immune system ^83– 85^. Induction of hyperammonemia in rats is associated with impaired neutrophil phagocytic activity leading to ROS production, thereby contributing to systemic inflammation and predisposing infections. Although the precise underlying mechanisms of the synergy between ammonia and inflammation in driving HE severity are not yet fully understood, it may provide essential novel therapeutic targets.

## Future perspectives on novel therapeutic targets

### Cornerstone pharmacotherapies: targeting the gut

The pharmacotherapies for the treatment of HE traditionally target the gut. The two main treatments in current routine clinical practice are non-absorbable disaccharides (i.e. lactulose) and the poorly absorbed antibiotic rifaximin ^1^. Lactulose is traditionally the first-line treatment in CLD-related HE. It reduces circulating ammonia levels by different mechanisms: 1) modulation of intestinal flora and therefore reduction in urease-producing bacteria and 2) its laxative effect reduces diffusion of ammonia and nitrogenous compounds into the bloodstream. Lactulose plays an important role as a first-line treatment of HE and in secondary prophylaxis of recurrent overt HE. A recent meta-analysis once again confirmed its beneficial effect regarding HE resolution, development of liver-related complications, and mortality ^86, 87^. These data, together with this treatment’s low cost and small spectrum of side effects, support the recommendation for lactulose as the initial therapy for HE in CLD. However, as a first-line treatment of an acute HE episode, there is no robust evidence that lactulose improves mortality and outcome. Rifaximin is derived from rifamycin and has a broad spectrum of action against Gram-positive, Gram-negative, aerobic, and anaerobic bacteria. Its mode of action is thought to be via modulation of the gut microbiota, thereby inducing a shift towards a less-pathogenic bacteria population and a reduction in ammonia, endotoxins, and pro-inflammatory cytokines. Addition of rifaximin to lactulose is recommended in patients with recurrent overt HE in CLD despite lactulose prophylaxis ^1^. In addition, several studies support the effectiveness of rifaximin in the setting of acute HE, but robust data showing improvement of survival in these patients are lacking ^88, 89^. Along with the globally increasing incidence of liver cirrhosis, the number of hospitalizations for HE has been continuing to grow over the last decade, despite the implementation of novel therapies, such as rifaximin ^2, 5^. Moreover, long-term antibiotic usage is associated with increased risk of infection with antibiotic-resistant strains of bacteria. Therefore, there is still a significant need for identifying and exploring novel therapeutic targets.

### Potential future therapies targeting the gut

Probiotics have been studied in a small number of trials that reported positive results regarding primary prevention of HE, risk of HE-related hospitalization, and the severity of liver disease ^90– 92^. As for rifaximin, the mechanism of action is thought to be through modulation of the gut microbiome and metabolism. However, it should be noted that probiotics have been reported to be unsafe in some categories of patients, such as those with acute pancreatitis ^93^. A report released by the World Health Organization (WHO)/Food and Agriculture Organization (FA) ( https://www.who.int/foodsafety/fs_management/en/probiotic_guidelines.pdf) describes four types of potential side effects, namely systemic inflammation, deleterious metabolic activities, excessive immune stimulation in susceptible individuals, and gene transfer. A more recent report from the Agency for Healthcare Research and Quality (AHRQ) reviews the results of 622 studies and concludes that, based on the currently available literature, questions on the safety of probiotics cannot be entirely addressed ^94^. Another novel, upcoming approach to restore the gut dysbiosis is fecal microbiota transplantation (FMT). In pre-clinical studies, it has been proven that transplantation of fecal microbiota can effectively reduce ammonia levels ^95, 96^. A small number of clinical trials have shown promising results in terms of the safety/tolerability profile and efficacy (hospitalization, cognition, dysbiosis) of FMT ^97, 98^. However, a recent case report describes two patients included in two independent clinical trials (one of them had advanced CLD and refractory HE), who developed extended-spectrum beta-lactamase (ESBL)-producing *Escherichia coli* bacteremia after they had received FMT oral capsules derived from the same stool donor ^99^. Therefore, well-selected stool transplantation may be an attractive future target for the treatment of HE, but continuous research on the risks and benefits of FMT and the optimization of donor screening is needed.

### Targeting ammonia and nitrogen metabolism

L-ornithine L-aspartate (LOLA) is a combination of two non-essential amino acids that can promote ammonia detoxification by acting as a substrate for the urea cycle and by activating GS ^100^. Intravenous administration has been reported to effectively reduce ammonia and improve mental state in overt HE, whereas the oral formulation seems effective in mHE by improving the outcome of psychometric tests ^101^. However, a recent Cochrane review states that the quality of evidence on the use of LOLA in HE is poor and further randomized controlled trials are needed ^102^. Therefore, administration of LOLA is currently restricted to countries in which it is approved for the treatment of HE. A potential risk of LOLA is the so-called “rebound” of ammonia production, as glutamine can be recycled into ammonia by glutaminases ^103^. The ammonia scavenger ornithine phenylacetate (OP) bypasses this issue by conjugating glutamine with phenylacetate, which forms a water-soluble molecule that can be excreted by the kidneys and hence prevents re-metabolism via glutaminase to glutamate and ammonia. OP was studied up to phase IIb trials and has been shown to safely and effectively reduce ammonia with a dose-related clinical improvement ^104– 106^. A planned phase III trial needs to confirm these findings.

Nitrogen scavengers that have been investigated in HE include sodium benzoate, glycerol phenylbutyrate, and sodium phenylbutyrate. These agents decrease ammonia by activating conjugation reactions, thereby promoting the elimination of waste nitrogen as amino acid conjugates instead of urea. Benzoate conjugates with glycine to form hippurate and phenylacetate with glutamine to form phenylacetylglutamine, which are both readily excreted by the kidneys ^107, 108^. Several trials have been showing a beneficial effect of these scavengers on ammonia levels and the risk of overt HE development ^109^. However, in a recent Cochrane review, it was concluded that the number of available studies is low and the quality of evidence poor at present ^109^. Furthermore, the high salt load required for sodium benzoate may imply a limited utility in the treatment of HE in CLD. Another approach that has been studied to reduce brain glutamine in HE is the inhibition of GS, for example by L-methionine-S,R-sulfoximine (MSO). Several pre-clinical studies have shown a beneficial effect of MSO on ammonia-induced astrocyte swelling and intracranial hypertension ^110– 114^. However, the translation of MSO to the clinical situation is hampered by the fact that it can cause convulsions, as observed in animal models ^115, 116^.

Finally, nutritional supplements, such as branch-chained amino acids (BCAAs) and zinc, have been studied. In the setting of impaired hepatic ammonia metabolism in CLD, skeletal muscle plays an important role in ammonia detoxification through glutamine synthesis by GS ^117, 118^. In cirrhosis, BCAAs are consumed in skeletal muscle to form α-ketoglutarate, which may be depleted due to enhanced amination to glutamate and subsequent glutamine synthesis ^119– 121^. Clinical trials assessing BCAA supplementation for the treatment of HE showed mixed results and further studies are required ^122– 126^. Nevertheless, BCAA supplementation is safe and seems to be useful in preventing the deterioration of liver failure and nutritional status of the cirrhotic patient ^127, 128^.

Zinc, often deficient in CLD, is considered a cofactor of urea cycle enzymes, and low levels are associated with hyperammonemia and HE ^129– 132^. The potential benefit of zinc supplementation has been studied in several clinical trials showing largely beneficial effects in HE ^133– 135^. A recent meta-analysis concluded that a combination of zinc with lactulose over 3–6 months may improve the outcome of psychometric tests in patients with covert HE as compared to lactulose alone ^136^.

### Targeting systemic inflammation

The concept of the beneficial effect of anti-inflammatory agents on HE has initially been shown for ibuprofen (NSAID) and indomethacin (a potent inhibitor of cyclooxygenase-2) in pre-clinical models of HE ^83, 137– 139^. However, it needs to be considered that these non-steroidal anti-inflammatories are not indicated in the context of CLD because of their deleterious effects on kidney function and impact on risk of gastrointestinal bleeding. Currently, the majority of studies are focusing on reducing the degree of endotoxemia and systemic inflammation by gut-targeting therapies as described above. Also, albumin administration has been studied as a treatment for HE. Besides its property to promote the maintenance of systemic oncotic pressure, albumin is able to scavenge toxins, has anti-oxidant properties, and stabilizes endothelial function ^140^. However, albumin was found to be ineffective in improving HE severity, ammonia levels, and markers of oxidative stress and inflammation in clinical trials, but it prolonged survival ^141, 142^. However, although no survival benefit was reported when albumin was applied in extracorporeal liver assist devices (i.e. MARS), it was associated with a decline in HE severity ^143^. This dialysis system may have its indication in HE as a bridge to transplantation or spontaneous clinical improvement in specialized centers.

A few approaches have been studied to target neuroinflammation directly. N-methyl-D-aspartate (NMDA) receptors are known to play an important role in some types of learning. The function of these receptors is reduced in models of chronic hyperammonemia ^144^. Activation of these receptors increases calcium in postsynaptic neurons, thereby increasing NO and cyclic guanine monophosphate (cGMP). This so-called glutamate–NO–cGMP pathway plays a key role in inhibiting neuroinflammation and promoting neural cell survival. Sildenafil is a phosphodiesterase inhibitor that inhibits the degradation of cGMP and thereby improves the function of the glutamate–NO–cGMP pathway. Sildenafil has been shown to improve learning abilities in rodent models of minimal HE. In addition, it has been reported to reduce neuroinflammation ^145– 147^. Targeting the NMDA receptors directly is clinically difficult because in acute hyperammonemia (in contrast to its behavior during chronic hyperammonemia) the NMDA receptors are highly activated and account for ammonia-induced mortality ^144^. Other studied approaches to reduce neuroinflammation involve reducing microglial activation directly by inhibiting the p38 mitogen-activated protein kinase ^148, 149^. Agusti *et al*. showed that inhibiting p38 reduces neuroinflammation (microglial activation and inflammatory markers) and improves cognitive and motor function (learning ability, motor activity, and coordination) in rats with portal systemic shunt-induced minimal HE ^150^. Current and potential therapies for HE in CLD and their recommended or studied doses are summarized in Table 2.

**Table 2.** Overview of the discussed (potential) treatment options for HE in CLD and their recommended or studied doses.

Treatment	Drug	Recommended/studied dose
Non-absorbable disaccharides	Lactulose	Initial dose 25 mL. Dose titration to maintain 2–3 loose bowel movements/day.
Antibiotics	Rifaximin	550 mg BD orally
Ammonia/nitrogen scavengers	LOLA	25–40 g/day (i.v.)
	OP	Up to 20 g/day (i.v.)
	Sodium benzoate	10 g/day (oral)
	Sodium phenylbutyrate	200 mg/kg/day (oral or via nasogastric tube)
	Glycerol phenylbutyrate	6 mL twice daily for 16 weeks (oral)
Albumin dialysis	MARS	various
Probiotics	various	various
FMT	n.a.	various
BCAAs	n.a.	various (13.2–60 g/day)
Zinc	n.a.	various (50–600 g/day)
Selection of experimental therapies targeting neuroinflammation	Indomethacin	0.5 mg/kg i.v.
	Sildenafil	unknown
	SB239063 (MAP-kinase-p38 inhibitor)	unknown

BCAA, branched-chain amino acid; BD, twice daily; FMT, fecal microbiota transplantation; HE, hepatic encephalopathy; i.v., intravenously; LOLA, L-ornithine L-aspartate; OP, ornithine phenylacetate.

## Conclusion

**Figure 1.**
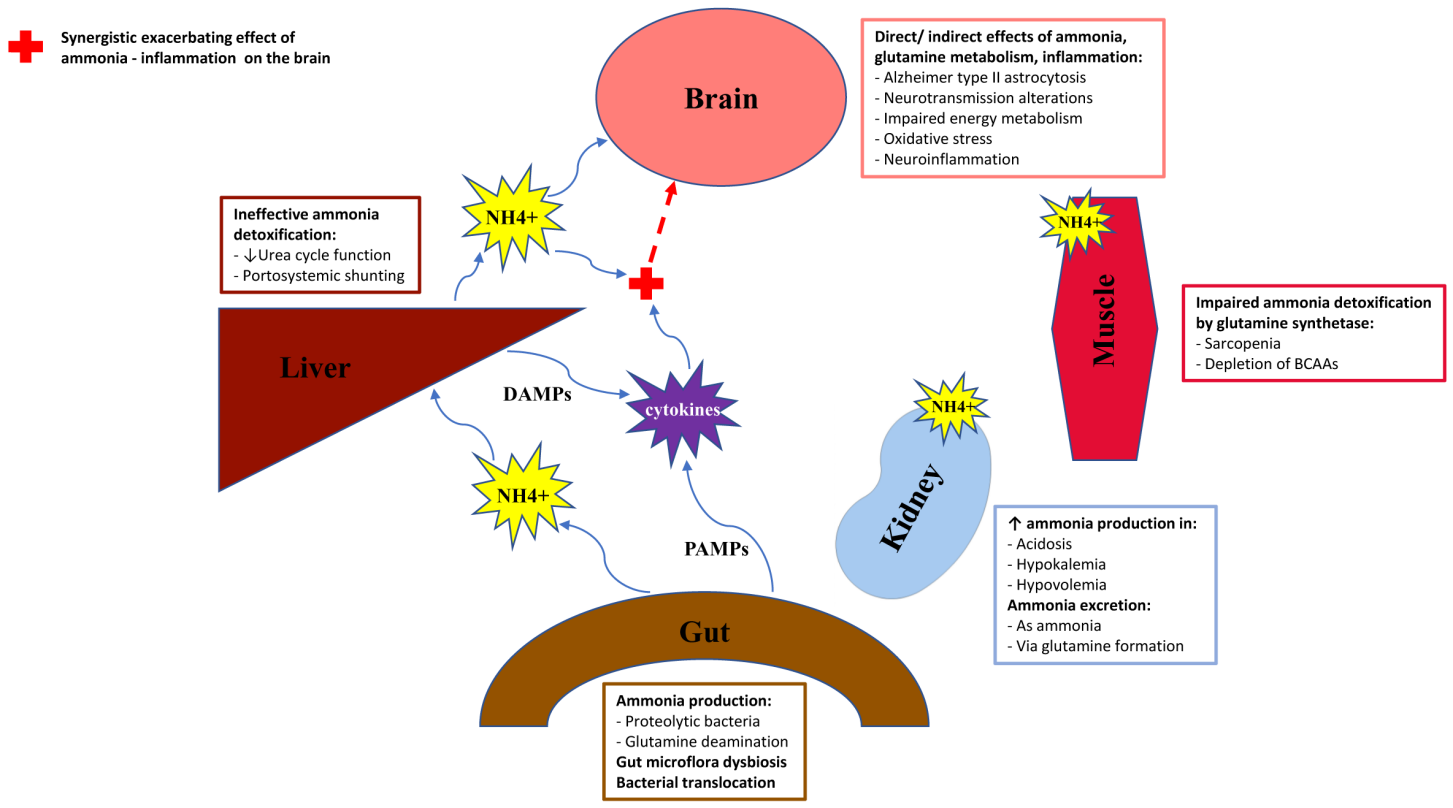
Multi-organ mechanisms of ammonia production and metabolization and its interaction with inflammation in the pathogenesis of hepatic encephalopathy. BCAAs, branched-chain amino acids; DAMPs, damage-associated molecular patterns; PAMPs, pathogen-associated molecular patterns.

HE is a devastating complication of end-stage liver disease with an ever-persisting impact on morbidity, hospitalization, and mortality. Currently available therapies in clinical practice are limited, and the development of novel approaches has been hampered by the complexity and heterogeneity of the syndrome. Traditionally, elevated levels of the neurotoxin ammonia have been considered to be the key driver of “portal-systemic HE”. In the last few decades, the involvement of extrahepatic organs in ammonia metabolism and the role of systemic and neurological inflammation in the pathogenesis of HE have been more and more established and gained their role in clinical management ( Figure 1). The development of novel therapies is currently mainly focusing on scavenging ammonia and modulating the gut microbiome, which are attractive potential treatment approaches. The synergistic relationship between ammonia and inflammation in modulating the severity of HE needs to be further explored, as it might provide us with effective novel therapeutic targets, especially in the setting of an acute bout of HE in CLD.

## Abbreviations

BBB, blood–brain barrier; BCAA, branch-chained amino acid; CBF, cerebral blood flow; cGMP, cyclic guanine monophosphate; CLD, chronic liver disease; ETC, electron transport chain; FMT, fecal microbiota transplantation; GS, glutamine synthetase; HE, hepatic encephalopathy; LOLA, L-ornithine L-aspartate; LPS, lipopolysaccharide; mHE, minimal HE; MSO, L-methionine-S,R-sulfoximine; NMDA, N-methyl-D-aspartate; NO, nitric oxide; OP, ornithine phenylacetate; PAMP, pathogen-associated molecular pattern; PTP, permeability transition pore; ROS, reactive oxygen species; TCA, tricarboxylic acid.
